# Selecting an anti-malarial clinical candidate from two potent dihydroisoquinolones

**DOI:** 10.1186/s12936-021-03617-1

**Published:** 2021-02-19

**Authors:** Yizhe Chen, Fangyi Zhu, Jared Hammill, Gloria Holbrook, Lei Yang, Burgess Freeman, Karen L. White, David M. Shackleford, Kathleen G. O’Loughlin, Susan A. Charman, Jon C. Mirsalis, R. Kiplin Guy

**Affiliations:** 1 Department of Pharmaceutical Sciences, University of Kentucky College of Pharmacy, Lexington, KY 40536 USA; 2 Department of Chemical Biology and Therapeutics, St. Jude Children’s Research Hospital, Memphis, TN 38105 USA; 3 Preclinical Pharmacokinetics Shared Resource, St. Jude Children’s Research Hospital, Memphis, TN 38105 USA; 4 Centre for Drug Candidate Optimisation, Monash Institute of Pharmaceutical Sciences, Monash University, Parkville, VIC 3052 Australia; 5 Toxicology and Pharmacokinetics, SRI International, Menlo Park, CA 94025 USA

**Keywords:** Candidate selection, Physicochemical properties, In vitro and in vivo metabolism, Bioavailability, Dose proportional exposure

## Abstract

**Background:**

The ongoing global malaria eradication campaign requires development of potent, safe, and cost-effective drugs lacking cross-resistance with existing chemotherapies. One critical step in drug development is selecting a suitable clinical candidate from late leads. The process used to select the clinical candidate SJ733 from two potent dihydroisoquinolone (DHIQ) late leads, SJ733 and SJ311, based on their physicochemical, pharmacokinetic (PK), and toxicity profiles is described.

**Methods:**

The compounds were tested to define their physicochemical properties including kinetic and thermodynamic solubility, partition coefficient, permeability, ionization constant, and binding to plasma proteins. Metabolic stability was assessed in both microsomes and hepatocytes derived from mice, rats, dogs, and humans. Cytochrome P450 inhibition was assessed using recombinant human cytochrome enzymes. The pharmacokinetic profiles of single intravenous or oral doses were investigated in mice, rats, and dogs.

**Results:**

Although both compounds displayed similar physicochemical properties, SJ733 was more permeable but metabolically less stable than SJ311 in vitro. Single dose PK studies of SJ733 in mice, rats, and dogs demonstrated appreciable oral bioavailability (60–100%), whereas SJ311 had lower oral bioavailability (mice 23%, rats 40%) and higher renal clearance (10–30 fold higher than SJ733 in rats and dogs), suggesting less favorable exposure in humans. SJ311 also displayed a narrower range of dose-proportional exposure, with plasma exposure flattening at doses above 200 mg/kg.

**Conclusion:**

SJ733 was chosen as the candidate based on a more favorable dose proportionality of exposure and stronger expectation of the ability to justify a strong therapeutic index to regulators.

## Background

The protozoan parasites of the *Plasmodium* family cause malaria, a disease affecting roughly 220 million patients and killing 405,000 people in 2018. Although the global eradication campaign has led to mortality falling by roughly 30% since 2010 (from 585,000 deaths to 400,000), the rate of decline has stalled since 2014, and even reversed in some areas [1]. Even though several vaccines have been developed, only one has shown efficacy with a partial positive effect in 55% of treated children [2]. Therefore, drug treatment remains a key part of any eradication campaign, in combination with mosquito control using sleeping nets and insecticides [3]. Artemisinin–based combination therapies (ACTs) are currently the standard of care for uncomplicated malaria [4]; however, acquired resistance to the individual components of ACTs has been rising [1, 5]. Although this resistance can currently be overcome with longer treatment schedules and/or higher doses [6], this situation raises the potential to return to an era when there are no antimalarials for which resistance does not exist. Therefore, the development of new drugs acting through novel modes of action is urgently needed to back up the ACTs.

The Medicines for Malaria Venture (MMV) maintains a set of Target Product Profiles (TPPs) and Target Candidate Profiles (TCPs) that define ideal antimalarial new drugs [7]. The ideal malaria drug would possess the following characteristics: good oral bioavailability, potency and duration of action sufficient to require only a single dose, rapid parasite clearance, a prolonged half-life to ensure clearance of any residual parasites, minimum risk of drug-drug interactions, ability to clear latent liver disease, and ability to block transmission. The number of compounds currently in the pipeline that at least partially satisfy this TCP has increased in the last decade [8], although a drug with strong potential for single dose cure is still lacking in late stage clinical trials. The expectation that new drugs will be combination medicines and the possibility of failure during clinical development demand that the discovery of candidates be an ongoing endeavour.

**Fig. 1:**
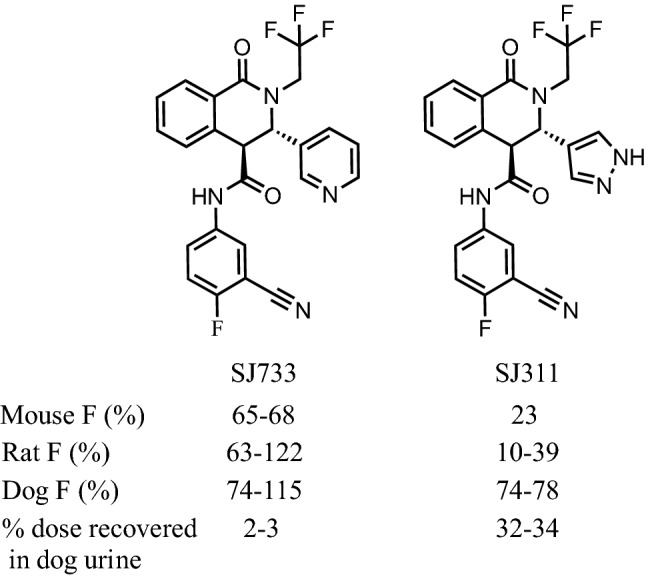
Structures of SJ733 and SJ311. F: Bioavailability

Studies leading to the discovery of (+)-SJ733 [9], which has recently completed Phase 1 trials, were previously disclosed [10]. (+)-SJ733 is the second inhibitor of PfATP4, a parasite proton-sodium antiporter, that has entered clinical trials—the other being cipargamin [11–13]. It has previously been shown that PfATP4 inhibitors selectively induce eryptosis of infected red blood cells leading to a rapid clearance of infected erythrocytes in vivo [9]. Although substantial work has subsequently been completed with SJ733, how it was selected as the clinical candidate over the sister late lead compound, SJ311, has not previously been described (Fig. 1). Herein, the physiochemical properties, pharmacokinetic, and toxicity profiles of both leads in multiple species are presented. Both compounds had desirable drug-like properties, however, SJ311 lacked dose proportionality in exposure in both mice and rats and possessed significant renal clearance in rats and dogs (renal clearance in mice was not assessed). These properties potentially limited the demonstrable safety margin for SJ311 relative to SJ733. Therefore, (+)-SJ733 was chosen as a clinical candidate.

## Methods

### Chemicals

Ethanol was purchased from DJ7656 Pharmco (Brookfield, CT); Propylene glycol (two lots used) from VWR (Brisbane, CA) and Avantor Performance Materials, Inc (Center Valley, PA); Carobowax Polyethylene glycol 400 (PEG 400) from Fisher Scientific (Hanover Park, IL); Kleptose HPB (Hydroxypropyl-B-cyclodextrin, Oral Grade (HPBCD) E0110) from Roquette America, Inc (Keokuk, IL); Phosphate buffered saline (PBS) from Invitrogen (Carlsbad, CA); Liver microsomes and NADPH-regenerating system from Xenotech (Kansas City, KS, and Corning (Tewksbury, MA,); Hanks’ balanced salt solution (HBSS) from Thermo Fisher Scientific (Waltham, MA); Caco-2 cells from American Type Culture Collection (Manassas, VA). Other reagents were commercially available and of special reagent grade, liquid chromatography mass spectrometry (MS) grade, or equivalent. (+)-SJ311 and (+)-SJ733 were provided by Dr. David Floyd’s group and synthesized according to the published route [14]. The only structural difference is exchange of a single pyridine (SJ733) ring for a pyrazole (SJ311).

### Animals

General procedures for animal care and housing were in accordance with the National Research Council (NRC) Guide for the Care and Use of Laboratory Animals, 8th edition (2011) and the Animal Welfare Standards incorporated in 9 CFR Part 3, 1991.

All murine studies were performed at St Jude Children’s Research Hospital (SJCRH) using C57BL6 mice 8 weeks of age or older (17–23 g). All mice were maintained in a temperature-controlled environment on a fixed 12-h light/dark cycle with free access to water and food. Studies were performed in strict accordance with the protocol approved by the SJCRH IACUC (Institutional Animal Care and Use Committee).

Rat studies performed at the Centre for Drug Candidate Optimisation (CDCO), Monash University (Australia), used male Sprague Dawley rats 8–9 weeks of age (267–291 g). Studies were conducted using established procedures in accordance with the Australian Code of Practice for the Care and Use of Animals for Scientific Purposes, and the study protocols were reviewed and approved by the Monash Institute of Pharmaceutical Sciences Animal Ethics Committee. Rat studies performed at the SRI International used male Sprague Dawley rats 8–9 weeks of age (220–317 g) with jugular vein catheterization performed by the Charles River Laboratories. Studies were performed in accordance with the protocol approved by the SRI IACUC.

Canine studies carried out at SRI used male beagle dogs 6–7 months of age (6.0–7.7 kg). Studies were performed in accordance with the protocol approved by the SRI IACUC.

### Solubility

#### Kinetic solubility

Solubility assays were carried out on a Biomek FX lab automation workstation (Beckman Coulter, Inc., Fullerton, CA) using µSOL Evolution software (pION Inc., Woburn, MA). Compound stock (10 mM in DMSO, 10 µL) was added to 1-propanol (190 µL) to make a reference stock plate. Reference stock solution (5 µL) was mixed with 1-propanol (70 µL) and citrate phosphate buffered saline (75 µL) to make the reference plate and the UV spectrum (250 nm–500 nm) of the sample plate was read. Test compound stock (10 mM in DMSO, 6 µL) was added to buffer (594 µL) in a 96-well storage plate and mixed. The storage plate was sealed and incubated at room temperature for 18 h. The suspension was then filtered through a 96-well filter plate (pION Inc., Woburn, MA). Filtrate (75 µL) was mixed with 1-propanol (75 µL) to make the sample plate, and the UV spectrum (250 nm – 500 nm) of the sample plate was read. Calculation was carried out by µSOL Evolution software based on the AUC_inf_ (area under curve) of UV spectrum of the sample plate and the reference plate. All compounds were tested in triplicate.

#### Thermodynamic solubility

The solubility of (+)-SJ733 was evaluated at 37 °C under neutral (isotonic phosphate buffer, ionic strength of 154 mM, pH 7.4) and acidic (0.1 N HCl, pH 1.0) conditions. Solubility was also evaluated in fasted (FaSSIF-V2) or fed (FeSSIF-V2) state simulated intestinal fluids. These media contain lipolysis breakdown products (glycerol monooleate and oleic acid) in addition to bile salt (sodium taurocholate) and phospholipid (lecithin) [15] and were buffered to simulate approximate pH conditions found in the fasted (pH 6.5) or fed (pH 5.8) state small intestine. Control media (blank FaSSIF-V2 and FeSSIF-V2 buffers) lacking bile salt, phospholipid and lipolysis products were also investigated.

Compounds were weighed into individual screw cap polypropylene tubes and aqueous buffer, 0.1 N HCl, or simulated intestinal fluid added to provide a compound concentration of between 700 and 5000 μg/mL. Samples were vortexed, placed in a 37 °C incubator, and mixed on an orbital shaker (IKA® VXR basic Vibrax® orbital shaker) set at 600 rpm. Samples were regularly examined to ensure excess solid was present. Sampling was conducted after 1, 4, 6, and 24 h by centrifuging each sample at 10,000 rpm for 3 min, transferring a 200 µL aliquot into fresh Eppendorf tubes, and centrifuging again at 10,000 rpm for 3 min. Duplicate aliquots of the final supernatant were removed and diluted to an appropriate analytical concentration in 50% aqueous methanol prior to analysis by HPLC. HPLC analysis was conducted on a Waters 2695 HPLC system coupled to a Waters 2487 dual absorbance wavelength detector, analyzing at 254 nm. A Phenomenex Luna C18(2) column (3 µm, 50 × 2.0 mm i.d.) was used for analysis, with the column temperature maintained at 40 °C. Concentrations were quantified by comparison to a calibration curve prepared over the concentration range in 50% aqueous methanol. The mobile phase consisted of water, methanol, and 1% aqueous formic acid. Separations were conducted using a flow rate of 0.4 mL/min and an injection volume of 5 µL. Processed samples were maintained in the autosampler at a temperature of 10 °C.

### Permeability

#### Parallel artificial membrane permeability assay (PAMPA)

The assay was conducted using a Biomek FX lab automation workstation (Beckman Coulter, Inc., Fullerton, CA) with PAMPA evolution 96 command software (pION Inc., Woburn, MA). Test compound stock (10 mM in DMSO, 3 µL) was mixed with citrate phosphate buffered saline (597 µL) to make diluted test compound. Diluted test compound (150 µL) was transferred to a UV plate (pION Inc., Woburn, MA) and the UV spectrum (250–500 nm) was read as the reference plate. Each well of the donor plate in a PAMPA sandwich plate (pION Inc., Woburn, MA) contained a filter that was painted on one side with 4 µL GIT lipid (pION Inc., Woburn, MA) to form a membrane. Each well in the acceptor plate in a PAMPA sandwich, preloaded with magnetic stir bars, was filled with acceptor solution buffer (200 µL, pION Inc., Woburn, MA). The donor plate was filled with diluted test compound (180 µL). The combined PAMPA plate was placed on a pIon Gut-box™ and stirred for 30 min. The UV spectrum (250–500 nm) of the donor and the acceptor were read. The permeability coefficient and recovery were calculated using PAMPA evolution 96 command software (pION Inc., Woburn, MA) based on the whole spectrum measured from the reference plate, the donor plate, and the acceptor plate. All compounds were tested in triplicate.

#### Caco-2 permeability

Caco-2 cells were maintained at 37 °C in a humidified incubator with an atmosphere of 5% CO_2_. The cells were cultured in Eagle's Minimum Essential Medium (EMEM) containing 20% fetal bovine serum (FBS) in 75 cm^2^ flasks, supplemented with 100 units/ml of penicillin and 100 µg/mL of streptomycin. The Caco-2 cells were seeded onto inserts of a 96-well plate (HTS-Transwell inserts, surface area: 0.143 cm^2^, Corning) at a cell density of 0.5 × 10^5^ cells/insert. The culture medium was replaced every 2 days, and the cells were maintained for 7 days at 37 °C and 5% CO_2_. Caco-2 cell monolayers with trans epithelial electrical resistance (TEER) values greater than 400 Ω cm^2^ were used for the subsequent assays. The permeability assay was initiated by adding an appropriate volume of HBSS/HEPES containing test compounds to either the apical (A to B) or basolateral (B to A) side of the monolayer, then adding the blank HBSS/HEPES buffer in the receiving compartment, the basolateral or apical side of the monolayer. The Caco-2 cell monolayers were then incubated for 2 h at 37 °C. To make a sample plate, fractions were collected from the basolateral side or apical side and quenched by adding onefold volume of acetonitrile with internal standard (2 µM warfarin) to each well. In a reference plate, the above HBSS/HEPES buffer containing test compounds were diluted with quenching solvent the same as that in the sample plate. 10 µL of supernatants were injected and analysis by UPLC/MS (Waters; Milford, MA). The test compound concentrations were quantified by comparing the sample well to the reference well via peak areas. The A→B (or B→A) apparent permeability coefficients (Papp, 10^–6^ cm/s) of each compound were calculated using the equation, Papp = dQ/dt × 1/AC_0_. The flux of a drug across the monolayer was dQ/dt (µmol/s). The initial drug concentration on the apical or basolateral side was C_0_ (µM). The surface area of the monolayer was A (cm^2^). The efflux ratio is the ratio of apparent permeability for a test compound in the basolateral to apical (B → A) direction relative to that in the A → B direction. All compounds were tested in triplicate.

### LogP, LogD, and pKa

#### LogD and pKa

Octanol/pH 7.4 buffer partitioning experiments were conducted using a shake flask method, and pKa was assessed by potentiometric titration. Both methods have been described previously [16].

#### LogP

LogP was measured using a Gemini Profiler instrument (pION Inc., Billerica, MA). 1–2 mg of compound was dissolved in octanol (0.5 mL) in a test tube. The test tube was purged with argon and a magnetic stir bar was added. The solution was treated with aqueous KCl (2.5 mL, 0.15 M) and stirred for 10 min. The pH was adjusted to 2 by addition of aqueous HCl (0.5 M). The resulting solution was titrated by adding aqueous KOH (0.5 M) in small aliquots (controlled by the Gemini Profiler software), until the pH reached 12. The volume of each addition and the corresponding pH of the test solution were recorded. Data were processed using pS software. The data points were fitted to a Bjerrum plot to achieve the best GOF (goodness of fitness) and a logP value was obtained. All measurements were conducted in triplicate.

### Stability in SGF (simulated gastric fluid) and CPBS

Compound stocks (10 mM in DMSO) were diluted to 2 mM in DMSO. The positive control was chlorambucil (10 mM in DMSO) and the internal standard was warfarin (2 µM in methanol). Freshly prepared simulated gastric fluid (0.4 g NaCl, 0.64 g pepsin, 1.4 mL concentrated HCl, 198 mL DI water) and citrate phosphate buffered saline (CPBS, pH 3, 5, and 7.4) (1.9 mL) were added to the wells of a master plate (2 mL 96-well deep well plate, pION Inc., MA, #110023). Chlorambucil (3.8 µL) or diluted compound solutions (3.8 µL, 2 mM) were added to each well and mixed. 600 µL of mixed solution was then removed from each well into two new wells to make triplicates. From the master plate, 65 µL of each sample was transferred into each of 8 storage plates (pION Inc., MA) allowing for eight time points. The storage plates were then incubated at 37 °C while shaking at 60 rpm. Stability was assessed at 0 min, 30 min, 1 h, 2 h, 4 h, 8 h, 24 h and 48 h by quenching the reaction with 195 µL of chilled methanol containing the internal standard, centrifuging at 4000 rpm for 15 min, and analyzing the supernatant by UPLC-MS. The compound and internal standard were detected by selected ion recording (SIR). Quantification of compound degradation was measured as a ratio to the internal standard and log peak area ratio was plotted as a function of time (h). Using the slope from the linear portion of this curve, the degradation rate constant was calculated. The rate constant was then used to calculate the half-life in SGF or CPBS.

### Plasma stability

Plasma stability assays were conducted in the same way as those of SGF/CPBS, except that three concentrations of compounds were prepared in DMSO:acetonitrile (1:4, v:v) at high (2 mM), medium (0.4 mM) and low (0.08 mM) concentrations. 1.9 mL each of mouse (Fisher Scientific, catalog #: NC9050370), rat (Fisher Scientific, catalog #: 50-415-345), dog (Fisher Scientific, catalog #: 50-415-573) or human plasma (Innovative Research Inc., catalog # IPLA-1) was added to wells, transferred, and analyzed the same way as those in the SGF and CPBS stability assay. The degradation rate constant and half-life in plasma were also calculated accordingly.

### Protein binding

A Rapid Equilibrium Dialysis (RED) Plate (Thermo Scientific, catalog #, PI-90007) was used to determine the percentage of compound binding to plasma proteins. The positive control for this experiment was propranolol-HCl (10 mM in DMSO) and the internal standard was warfarin (2 µM in methanol). 10 mM stocks of compound in DMSO were diluted with DMSO acetonitrile (1:4, v:v) to three different intermediate concentrations: high (2 mM), medium (0.4 mM) and low (0.08 mM). A 10 mM stock of propranolol in DMSO was diluted to 0.4 mM concentration in DMSO:acetonitrile (1:4 v:v). In 16 Eppendorf tubes, the control (10 µL) or each of three compound dilutions (10 µL) were each added to separate aliquots of mouse, rat, dog, and human plasma (990 µL). In the RED plate, potassium phosphate buffer (500 µL, 0.1 M, pH 7.4, 37 °C) was placed in every white well and each plasma/compound mixture was added to each of 3 red wells. The RED plate holds triplicate samples of one control (final concentration 0.4 µM) and one compound (final concentrations: 20 µM, 4 µM, 0.8 µM). The RED Plate was sealed and incubated at 37 ºC with shaking at 60 rpm for 4 h. The changes of pH value over the course of incubation was less than 0.1. After incubation, aliquots (50 µL) from each well in the RED plate were transferred to an assay plate. In order to create a uniform matrix in every well of the assay plate, plasma (50 µL) was added to each of the wells that already contained buffer and potassium phosphate buffer (50 µL) was added to each of the wells that already contained plasma/compound. Pre-cooled internal standard (300 µL) was added to the assay plate to quench the reaction. The compounds and internal standard were detected by selected ion recording (SIR). Using the peak area ratio of compound to warfarin from the SIR spectra, the percentage of free compound [1] and bound compound [2] were calculated using the following equations: (1) % free = (concentration buffer chamber/concentration plasma chamber)*100, and (2) % bound = 100 − % free.

### Whole blood-plasma partitioning

Human whole blood, collected using heparin as anticoagulant, was procured from the Volunteer Blood Donor Registry (Walter and Eliza Hall Institute of Medical Research) and used on the day of collection. The hematocrit (Hct) determined by centrifugation (13,000×*g* for 3 min using a Clemets® Microhematocrit centrifuge and Safecap® Plain Self-sealing Mylar Wrapped capillary tubes) was 42%. Blood to plasma partitioning was determined as previously described [16].

### Microsomal stability

The metabolic stability assay was performed by incubating compounds individually (0.8, 4, or 20 µM) with mouse, rat, dog and human liver microsomes (Fisher, 0.5 mg/mL protein concentration) at 37 °C. The metabolic reaction was initiated by the addition of a NADPH-regenerating system and quenched at various time points by the addition of acetonitrile according to a published method [14].

The remaining concentration of each compound was measured as a ratio of peak area to the internal standard. The log peak area ratio was plotted vs. time (h), and the slope was determined to calculate the elimination rate constant [k = (− 2.303) * slope]. If deviation from first order kinetics was evident, only the initial linear portion of the plot was used to determine the rate constate, k. The half-life (h) was calculated as t1/2 = 0.693/k. Intrinsic clearance in vitro was calculated as CLint_*in vitro*_ = (1000)*(0.693/t1/2*60)/0.5, where microsomal concentration in the reaction solution was 0.5 mg/mL; 1000 and 60 are scaling factors for volume (µL) and time (min), respectively. The intrinsic in vitro clearance was scaled to the intrinsic in vivo clearance using physiology based scaling factor (PBSF): CLint _in vivo=_ CLint _in vitro_ *PBSF: (microsome protein/gram liver) * (gram liver/kg b.w.) [14, 17], with PBSF: 47 (mouse), 47(rat), 58 (dog), 32 (human); and liver weight proportions: 54.9 (mouse), 36.6 (rat), 32.9 (dog) 25.7 (human).

### Hepatocyte stability

SJ733 (1 µM; n = 2 replicates) was incubated at 37 °C with suspensions of human, dog, rat, and mouse cryopreserved hepatocytes (XenoTech, Lenexa, KS). The average viable cell concentration over the incubation period was determined by the Trypan Blue exclusion method (in the absence of test compound). At various time points over the 60 min incubation period, the incubation mixtures were quenched by addition of ice-cold acetonitrile containing 0.52 µM of diazepam as an internal standard. The relatively short incubation time of 60 min was used to ensure hepatocyte viability over the incubation period. The relative loss of parent compound was quantified by LC–MS using a Waters Micromass Xevo G2QTOF mass spectrometer against calibration standards prepared in pre-quenched (to inactivate enzymes) blank hepatocyte mixture. The lower limit of quantitation value for the assay was 0.039 µM.

Test compound concentration versus time data were fitted to an exponential decay function to determine the apparent first-order rate constant for substrate depletion (k) that was then used to calculate the degradation half-life and the in vitro intrinsic clearance [3]: *CLint* = (k/hepatocyte cell number (10^6^ viable cells / mL).

Each value for CL_*int, *in vitro_ was multiplied by a PBSF to obtain the predicted in vivo intrinsic hepatic clearance, CL_*int, *in vivo_ [17, 18]. The predicted in vivo blood clearance (predicted CL_*blood*_) was then obtained by application of the well-stirred model of hepatic elimination [4]: Predicted Blood CL = (Q*CLint vivo/Q + CLint vivo), where Q is the nominal hepatic blood flow. Binding to hepatocytes and plasma protein were not taken into account.

### Recombinant human cytochrome P450 (rhCYP) enzyme assays

SJ733 was first pre-incubated with Bactosomes™ (Cypex Ltd, final P450 concentration: CYP1A1 25 pmol/mL, CYP1A2 100 pmol/mL,CYP1B1 100 pmol/mL, CYP2B6 100 pmol/mL, CYP2C8 50 pmol/mL, CYP2C9, 25 pmol/mL, CYP2C19 100 pmol/mL, CYP2D6 50 pmol/mL and CYP3A4 25 pmol/mL, 0.1 M phosphate buffer pH 7.4) at 37 °C prior to the addition of NADPH (final concentration 1 mM) to initiate the reaction with a final incubation volume of 50 μL. Incubations were also performed using control Bactosomes™ (no P450 enzymes present) to reveal any non-enzymatic degradation. Control compounds known to be metabolized specifically by each P450 isoform were included individually. Test articles and controls were incubated with each isoform for 0, 5, 15, 30, and 45 min. The reactions were stopped by transferring 20 μL of the reaction mixture to 60 μL methanol at the appropriate timepoints.

In an alternate approach SJ311 was studied using a validated cocktail probe substrate method. SJ311 was incubated (500 μL, 10 μM) at 37 °C with a cocktail of two or three probe substrates at concentrations equal to their approximate Km values for human CYP enzymes (0.2 mg/mL human liver microsomes, 10 mM MgCl_2_, and 100 mM potassium phosphate buffer (pH 7.4). SJ311 was pre-incubated for 5 min in with the addition of NADPH regenerating system, followed by incubation for 10 min and terminated by addition of 0.5 mL acetonitrile containing 0.2 μM dextrorphan as an internal standard. The termination plates were centrifuged at 3400 rpm for 10 min at room temperature to precipitate the protein. All samples were analyzed using LC MS/MS, with either positive atmospheric pressure chemical ionization (APCI) mode (SJ311) or ESI (SJ733) mode utilizing multiple reaction monitoring (MRM) scans.

### In vivo PK studies

Studies were undertaken to determine the plasma pharmacokinetics of SJ733 and SJ311. These studies included: (i) single oral gavage (PO) or intravenous (IV) dose administration to female C57BL/6 mice; (ii) single PO or IV dose administration to male Sprague Dawley (SD) rats; (iii) single PO or IV dose administration to male beagle dogs; (iv) toxicokinetic (TK) study following single PO administration to male SD rats.

### Formulations

The PO and IV formulation used for mouse studies at SJCRH was 1% hydroxypropyl-beta-cyclodextrin (w/v), 10% ethanol (v/v), 10% propylene glycol (v/v), 40% PEG-400 (v/v) and 39% PBS (pH 7.4) isotonic (v/v). Compounds were dosed orally as suspensions, and intravenously as filtered solutions. Compound concentrations were confirmed post filtration using UV spectroscopy. The IV formulation used for rat studies (Monash University) was the same as that used for mice. For PO dosing to rats (Monash University) at 2 mg/kg, a suspension formulation was used containing 0.5% (w/v) hydroxypropyl methylcellulose, 0.5% (v/v) benzyl alcohol and 0.4% (v/v) Tween 80. The formulation used for rat studies at SRI (PO, high dose) was the same as that used in mouse PK studies. The formulation for the high dose TK study at SRI was 0.5% methylcellulose in sterile water. The formulation used for dog studies was the same as that used in mouse PK studies.

#### i) Mouse

The PO and IV PK of SJ733 and SJ311 were studied in female C57BL/6 mice. Mice had access to water and food ad libitum throughout the pre- and post-dose sampling period. Doses were administered at 15 mg/kg for IV and 10–200 mg/kg for PO with 20 mice in each dosage group. Two samples were taken from each mouse, with the first sample being a retro-orbital bleed (~ 200 µL) at the indicated time point (5, 15, 30 min, 1, 4, 24 h) and the second being terminal cardiac puncture (~ 500 µL) at the indicated time point (usually 48 h). EDTA disodium was used as anticoagulant and added to whole blood (10% volume of EDTA for 1% w/v final concentration) followed by centrifugation at 13,000 rpm for 2 min. Plasma was collected and stored frozen at -20 °C until analysis.

#### ii) Rat

The PO and IV PK of SJ733 and SJ311 were studied in overnight-fasted male Sprague Dawley rats. Rats had access to water ad libitum throughout the pre- and post-dose sampling period, and access to food was re-instated 4 h post-dose. Each compound was independently administered as a 10 min constant rate IV infusion (4.5–5.1 mg/kg, 1.0 mL per rat, n = 2–3 rats) through a cannula surgically implanted in the jugular vein on the day prior to dosing. Oral doses (1.9–21.3 mg/kg) were administered via gavage. Samples of arterial blood and total urine were collected up to 48 h post-dose. Once collected, blood samples were centrifuged, supernatant plasma was removed and stored frozen (− 20 °C) until LC–MS analysis within 1 week of collection.

The high dose levels of both compounds (50, 100, 200 mg/kg, PO, (+)-SJ733/311) were also independently tested by SRI, with a single oral gavage administration. Blood (through jugular vein) and urine were collected at time points up to 72 and 24 h post dose, respectively. Supernatant plasma was removed following centrifugation and stored frozen (− 70 °C) until LC–MS analysis. For toxicology studies, male Sprague Dawley rats were administered 50, 100, 250, 500, or 750 mg/kg of (r)-SJ733 or (r)-SJ311 by oral gavage (one dose per animal). Body weights were recorded on Day 1 prior to dose administration and on Day 4. Blood samples (400 µL) were collected at 4 and 24 h post drug administration from the retro-orbital sinus. Potassium EDTA treated plasma was collected and kept frozen at -70 °C for bioanalytical analysis.

#### iii) Dog

The plasma PK of SJ733 and SJ311 following a single PO gavage or IV dose (via saphenous vein) to male beagle dogs was determined at an IV dose of 3 mg/kg and PO doses of 3 and 30 mg/kg (n = 3 for each). Briefly, this PK study was carried out in three sessions with one week washout period in between to allow for complete clearance of compounds. In the first session, 6 male dogs were administered a single 3 mg/kg IV dose of SJ311 or SJ733. In the subsequent second and third sessions, the same 6 male dogs were given a single 3 mg/kg or 30 mg/kg PO dose of SJ311 or SJ733, respectively. Plasma (0 -72 h) and urine (0–48 h) samples were collected for further analysis of SJ311 or SJ733 concentrations.

### Bioanalytical methods

For studies at SJCRH, all blood samples were kept on wet ice after collection and processed to plasma within 30 min of collection. Plasma samples were kept on dry ice and transferred to ≤ − 20 °C until analysis. Mouse plasma samples were extracted via protein precipitation with cold acetonitrile. The detection of the SJ733, SJ311, and warfarin (IS) was conducted by LC–MS with SIR or LC–MS/MS with MRM detection. Aliquots (3 µL) were injected onto a Waters Acquity UPLC equipped with an ABI Sciex 6500 Qtrap MS/MS and separated using an Acquity BEH C18 column (50 × 2.1 mm, 1.7 µm) with a methanol–water gradient containing 0.1% formic acid.

For studies at Monash University, rat plasma and urine samples were extracted utilizing protein precipitation with a twofold volume ratio of acetonitrile. SJ733, SJ311, and diazepam (IS) were detected using LC–MS/MS instrumentation. Aliquots (3 µL) were injected onto a Waters Acquity UPLC equipped with a Waters Micromass Xevo TQ MS/MS and separated using a Supelco Ascentis Express RP Amide column (50 × 2.1 mm, 2.7 µm) with a methanol–water gradient containing 0.05% formic acid. Calibration standards were prepared by spiking blank matrix (plasma or urine) and the calibration range was from 1 to 10,000 ng/mL for plasma or 2.5 to 5000 ng/mL for urine.

Rat samples from the toxicokinetic study at SRI were analyzed at SJCRH using the method described above. The calibration range was from 47 to 26,000 ng/mL for plasma.

Plasma and urine samples from the high dose rat PK studies were analyzed by SRI. In both matrices, the sample volume was 50 μL, and assay entailed the addition of 50 μL of internal standard solution to the standards and study samples. The compounds were used as reciprocal standards for one another (427 nM SJ733 in Milli-Q-Water for SJ571311 and 437 nM SJ311 in Milli-Q-Water for SJ733). These mixtures were then extracted with 1000 μL of ethyl acetate by vortexing for 10 min on a multi-tube vortex mixer at maximum speed followed by separation of the organic and aqueous phases by centrifugation (18,000×*g*, 5 min). Eight hundred microliters of the ethyl acetate (upper) layer of each sample were transferred to a clean tube and evaporated in a centrifugal evaporator without the application of heat. The dried samples were reconstituted with 100 µL of 10/90 (v/v) acetonitrile/Milli-Q-Water solution containing 0.1% formic acid. The reconstituted samples were then vortexed for 5 min on a multi-tube vortex mixer at one quarter speed, clarified by centrifugation (18,000×*g*, 3 min), and transferred to HPLC vials fitted with glass inserts for LC–MS/MS analysis. Aliquots (10 µL) were injected onto a Waters 2795 Alliance LC and Waters Micromass Quattro Ultima MS/MS and separated using a Phenomenex Luna C18 column (30 × 3 mm, 5 µm) with 2-propanol-water gradient containing 20 mM acetic acid. Study samples were quantitated using a set of calibration standards prepared in blank matrix that were processed in parallel.

Dog plasma was extracted utilizing protein precipitation with a twofold volume ratio of acetonitrile, while urine samples were extracted utilizing liquid–liquid extraction with ethyl acetate. SJ733, SJ311, and verapamil (IS) were quantitated by LC–MS/MS as described above.

SJ733 and SJ311 were shown to be stable (± 15% variance) when stored at − 80 °C for more than 14 days. All plasma samples that were shipped elsewhere from the testing facility were all analyzed within the validated stability time period.

### Pharmacokinetic analysis

Plasma concentration time (Ct) data for SJ733 and SJ311 were grouped by nominal time point, and the mean Ct values were subjected to noncompartmental analysis (NCA) using Phoenix WinNonlin 8.1 (Certara USA, Inc., Princeton, NJ). For all experiments, the area under the Ct and first moment curves (AUC, AUMC) were estimated using the “linear up log down” method. The terminal phase was defined as at least three time points at the end of the Ct profile, and the elimination rate constant (K_el_) was estimated using an unweighted log-linear regression of the terminal phase. The terminal elimination half-life (T_1/2_) was estimated as 0.693/K_el_, and the AUC from time 0 to infinity (AUC_inf_) was estimated as the AUC to the last time point (AUC_last_) + C_last_ (predicted)/K_el_ with the AUC_inf_ similarly calculated. Additional parameters estimated included observed maximum concentration (C_max_), time of C_max_ (T_max_), concentration at the last observed time point (C_last_), time of C_last_ (T_last_), and the apparent oral terminal volume of distribution (V/F). The apparent oral clearance (CL/F), systemic clearance (CL) and volume of distribution at steady state (V_ss_) were all estimated using standard formulae [19].

## Results

### Physiochemical parameters, solubility, and permeability

Both compounds were stable in all media tested (PBS, plasma, SGF), at all pHs tested, for the full duration of the assay. The stability of these compounds in vitro predicts that they are likely stable in the GI tract.

The logD_7.4_ values of both compounds were in the range of 2–4 suggesting that they have moderate lipophilicity at neutral pH and therefore are likely to have reasonable absorption from the GI tract (Table 1). However, there is a significant difference between logD values of the two candidates, with SJ311 showing lower lipophilicity (2.3) than SJ733 (3.9).

**Table 1:** Summary of pKa, LogP, and LogD of SJ733 and SJ311

	(r)-SJ733	(+)-SJ733	(r)-SJ311		(+)-SJ311
pKa	10.9 ± 0.1; 4.06 ± 0.03^*a*^		11.1 ± 0.3		
LogP	2.95 ± 0.03 (logP( +))		1.24 ± 0.28 (logP( +))		
LogD _(7.4) Shake flask_		3.90 ± 0.01^a,b^	2.34		2.30

^a^Data from Charman et al. [16]

^b^Averaged from analysis of high and low concentration partition samples, the results of which were highly similar (< 0.01 difference)

The kinetic solubilities of both compounds were roughly equivalent at pH levels ranging from 3 to 7.4 (Table 2). The thermodynamic solubility of SJ733 (Additional file 1: Table S1) showed equilibration still occurring until the 4 h time point. In pH 7.4, 6.5, and 5.8 aqueous buffers, the solubility of SJ733 was moderate, with values ranging from 81 to 237 µM, roughly equivalent to what was seen with the kinetic measurements. Under strongly acidic conditions, the compound was very soluble (> 3 mg/mL) presumably due to protonation of the pyridine group (pKa = 4.1).

**Table 2:** Summary of kinetic solubility of (r)-SJ733 and (r)-SJ311

pH	Kinetic solubility (µM)	
	(r)-SJ733	(r)-SJ311
3	84 ± 3	80 ± 7
5	84 ± 3	80 ± 7
7.4	65 ± 4	66 ± 7

Data are shown as mean ± SD (n = 3)

The PAMPA permeability of SJ733 was higher under basic conditions but diminished significantly at lower pH, likely due to protonation of the pyridine ring. However, the permeability of SJ733 was consistently higher than that of SJ311 (Table 3), whose permeability was pH-independent. Both compounds were unlikely to be actively effluxed in the intestine, as the efflux ratios (1.2–1.3), calculated from the Caco-2 permeability assay, were far below the values of known effluxed substrates [20].

**Table 3:** Summary of Caco-2 and passive permeability (PAMPA) of SJ733 and SJ311

Method	pH	Permeability (10^–6^ cm/s)	
		(r)-SJ733	(r)-SJ311
PAMPA	3	66 ± 3.5	17 ± 2.3
	5	380 ± 7.2	15 ± 6.7
	7.4	350 ± 130	9.4 ± 7.0
Caco-2	Apical to Basal	9.4 ± 1.6	12.5 ± 0.7
	Basolateral to Apical	11.0 ± 1.6	16.6 ± 2.0
	Efflux Ratio	1.17 ± 0.04	1.34 ± 0.21

Data are shown as mean ± SD (n = 3)

### Microsomal and hepatocyte stability

The stability of both compounds was tested in the presence of hepatocyte derived microsomes from mouse, rat, dog, and human (Table 4). The rate of metabolism was not affected by cofactor-independent metabolism.

**Table 4:** Summary of microsomal half-life and clearance in vitro of SJ733 and SJ311

Species	Parameter	(-)-SJ733	(+)-SJ733	(−)-SJ311	(+)-SJ311
Mouse	t_1/2_ (h)	0.8 ± 0.1	0.7 ± 0.1	> 4	> 4
	CLint (µl/min/mg protein)	32.1 ± 2.1	35.4 ± 2.1	< 7	< 7
Rat	t_1/2_ (h)	> 4	> 4	> 4	> 4
	CLint (µl/min/mg protein)	< 7	< 7	< 7	< 7
Dog	t_1/2_ (h)	2.2 ± 0.4	> 4	> 4	> 4
	CLint (µl/min/mg protein)	10.9 ± 1.5	< 7	< 7	< 7
Human	t_1/2_ (h)	0.4 ± 0.1	0.5 ± 0.1	> 4	> 4
	CLint (µl/min/mg protein)	61.7 ± 13.2	45.5 ± 4.0	< 7	< 7

Compounds with a calculated half-life longer than 4 h were all reported as having a half-life of > 4 h, and a clearance value < 7 µl/min/mg protein. Compounds were tested at a concentration of 0.8 µM. Data were presented as mean ± SD (n = 3)

There was significant variation in the in vitro intrinsic clearance exhibited across species. Both compounds were most stable in rat microsomes. Both compounds were also quite stable in dog microsomes, although one isomer of SJ733, (−)-SJ733, showed moderate degradation. SJ733 was most rapidly metabolized in human microsomes (closely followed by mouse) and there was no significant variation among the isomers. SJ311 was significantly more stable to microsomal metabolism in both human and mouse microsomes, without significant variation among the isomers. In experiments where a range of compound concentrations were explored there was clear evidence of saturation of metabolism at higher compound concentrations (Additional file 1: Table S2). Changing the pyridine (in SJ733) to the pyrazole (in SJ311) is expected to remove the potential for phase I N-oxidation of the aryl ring, which could be reflected here.

The stability of (+)-SJ733, the pharmacologically active isomer, was also tested in the presence of viable suspensions of cryopreserved hepatocytes from the same four species (Additional file 1: Table S3). SJ733 exhibited low to moderate rates of degradation in human, dog, rat, and mouse hepatocytes. As with the microsomal models, the general trend was slowest metabolism in the rat and dog and more rapid metabolism in human and mouse. The intrinsic in vivo clearance values based on human hepatocytes (Additional file 1: Table S3) were consistent with those based on NADPH-dependent degradation data in microsomes (Additional file 1: Table S2). However, the hepatocyte-predicted values for rat and mice were higher than the predicted values determined in microsomes. The hepatocyte-predicted blood clearance in rats agreed well with the measured in vivo clearance. When corrected for expected liver blood flow, these results predicted very rapid metabolism in the mouse and moderately rapid and roughly equivalent metabolism in the other three species (Additional file 1: Table S3).

### rhCYP inhibition and metabolism

CYP1A2, 2C9, 2C19, 2D6, 3A4 are the five most common isoforms of the cytochrome P450 (CYP) enzyme family involved in drug metabolism, accounting for more than 90% of known metabolism of drugs [21]. Because inhibition of CYP450 enzymes poses potential risk of drug-drug interactions, both compounds were tested to determine if they inhibited these CYP isoforms. Both SJ733 and SJ311 were moderate inhibitors of CYP3A4 and weak inhibitors of CYP1A2, 2C9, and 2D6 (Table 5). SJ733 suppressed the activity of 2C9 by 25% and 3A4 by 38% at 10 µM, but weakly inhibited 1A2, 2C19, and 2D6. No time dependent inhibition was observed. SJ311 caused 53% inhibition of 3A4 at 10 μM but was a weak inhibitor of CYP1A2, 2C9, 2C19, and 2D6. Therefore, neither compound has a high risk of P450-driven drug interactions.

**Table 5:** Summary of inhibition of CYP450 by (r)-SJ733 and (r)-SJ311

CYP	(r)-SJ733	(r)-SJ311	Probe substrate	Probe substrate metabolite
CYP1A2	− 5, 10.8%^a^	5, 0.5%	Ethoxy-resorufin	Resorufin
CYP2C9	34, 17%	19, 15%	Diclofenac	4′-Hydroxy diclofenac
CYP2C19	5, 5%	12, − 2%	S-mephenytoin	4′-Hydroxy mephenytoin
CYP2D6	7, 9%	11, − 4%	Bufuralol	1′-Hydroxy bufuralol
CYP3A4	42, 33%	54, 52%	Midazolam	1′-Hydroxy midazolam

^a^Values in columns represent replicate measurements. Compounds were tested at 10 µM

The primary metabolism of SJ733 was studied. There was no detectable formation of metabolites after incubation with CYP1A1, 1A2, 1B1, 2B6, 2C9, or 2C19 recombinant enzymes. Metabolite formation was only observed in the incubations with CYP2C8, 2D6, and 3A4 recombinant enzymes, with a maximum peak area ratio observed at 45 min of incubation. The exact amount of the metabolite was not measured.

### Plasma protein and whole blood binding

Both compounds are moderately bound to plasma proteins derived from all species, with ranges from 88.3 to 95.7% (Table 6). The plasma proteins that bound SJ733 or SJ311 were not characterized and remain unknown.

**Table 6:** Plasma protein binding and whole blood partitioning of (r)-SJ733 and (r)-SJ311

Species	**(**r)-SJ733		(r)-SJ311	
	% bound^*a*^	B/P ratio	% bound^*a*^	B/P ratio
Mouse	95.7 ± 0.6		93.7 ± 0.7	
Rat	90.0 ± 1.7		91.0 ± 0.1	
Dog	88.3 ± 3.1		90.0 ± 1.0	
Human	94.3 ± 0.6	0.72 ± 0.02^b^	95.3 ± 0.6	

^a^Values of protein binding are average of all high, medium and low concentrations (20, 4, 0.8 µM), all of which were similar. Data are presented as mean ± SD (n = 3)

^b^Value reported previously [16]

### Single dose tolerability and pharmacokinetic experiments in mice, rats and dogs

Both compounds were subjected to in vivo dose ranging and pharmacokinetics experiments. No significant adverse events were observed with either test compound after a single dose in any species. Ruffled fur (a general sign of stress) was noted in the highest dose group in rats (750 mg/kg) for both compounds, as well as in the 100 and 500 mg/kg groups treated with (r)-SJ311. When studies included clinical chemistry or hematology monitoring, no significant changes were associated with compound administration in any parameter. Likewise, when gross or histopathology was performed, there were no significant compound associated changes. This profile suggested that safety would not be a determining factor in selecting one compound over the other for advanced development.

The pharmacokinetics of SJ733 and SJ311 were studied following a single PO or IV dose to female C57BL/6 mice. These studies included vehicle controls; an IV dose of 15 mg/kg (Additional file 1: Table S5); and PO doses of 10, 50, 100, and 200 mg/kg (Table 7). Non-compartmental analysis of compound plasma concentration data revealed the terminal half-life of each compound to be similar: SJ311 (1.38–1.56 h) and SJ733 (1.38–1.65 h). When administered orally, SJ733 exhibited rapid absorption and a high oral bioavailability (F: 65–98%) with some evidence of saturable absorption at higher doses (extended T_max_ and longer apparent half-life); half-life values at lower doses were similar to those of IV. On the other hand, SJ311 exhibited a much lower oral bioavailability (F: 23%) but otherwise similar parameters to the IV route.

**Table 7:** Murine plasma pharmacokinetic parameters of S733 and SJ311 after oral administration

Dose (mg/kg)	(r)-SJ733		( +)-SJ733		(r)-SJ311
	50	200	10	100	100
C_max_ (µM)	10.1 ± 4.5	12.7 ± 3.2	1.38 ± 0.21	11.7 ± 1.2	10.5 ± 5.3
T_max_ (h)	0.5	5	0.5	0.5	0.5
Half-life (h)	1.72	14.1	3.63	3.17	4.20
AUC_inf_ (h µM)	28	155.7	4.27	53	42
CL/F (L/h/kg)	4.02	3.25	5.00	4.02	5.34
V/F (L/kg)	18.4	3.23	26.2	18.4	32.4
F (%)	70	98	65	80.8	23.1

% F was calculated using the equation: (AUC_inf_ PO / mean AUC_inf_ IV) • (Dose IV / Dose PO). Cmax values are mean ± SD, n = 3 or 4. AUC_inf_, CL and V were estimated from mean plasma concentration values from different animals in a single study; error or SDs for the parameters were not estimated

The pharmacokinetics of SJ733 and SJ311 were also studied in rats following a single PO or IV dose. The IV half-lives of the isomers and racemate of SJ733 varied between 5 and 18 h (Additional file 1: Table S6) at similar doses. Consistent with the data in microsomes, clearance was somewhat lower in rats than in mice. The fraction of the IV dose recovered in urine over the 24-h sampling period was very low (0.35–1.1%) for (+)-SJ733, suggesting that direct urinary excretion is not a major in vivo clearance pathway. Overall, SJ311 had similar IV PK parameters; however, the fraction of SJ311 recovered in urine was significantly higher than SJ733 (ca. 22% vs 1.5%), suggesting that renal excretion is a significant clearance pathway for SJ311.

At an oral dose of 20 mg/kg in the rat, the T_max_ of (+)-SJ733  was ~ 3 h (Table 8). For SJ733, dose proportionality in absorption related parameters was generally observed over the dose range of 50–200 mg/kg (suspension formulation). But for SJ311 there was possible saturation of absorption at doses greater than 100 mg/kg (Table 8). SJ733 achieved higher exposure at all dose levels than did SJ311 (mean AUC_inf_ = 96.3–330 vs. 34.1–65.8 h.µM). The terminal half-life of SJ733 was somewhat longer than for SJ311 (mean t_1/2_ = 7.7–10.3 h vs. 3.5–7.9 h). Both compounds showed fairly high volumes of distribution (mean V_ss_ 2.2–5.4 L/kg) and moderate clearance (14–25% of hepatic blood flow). Contrary to the IV dose, recovery of unchanged SJ311 was reduced at the high oral dose(s), suggesting a possible role of renal transporters, inferred from an apparent saturable elimination process. The effects of suspension vs. solution dosing were also explored for SJ733. The AUC_inf_ after solution doses were approximately threefold higher at 2 mg/kg and twofold higher at 20 mg/kg than that observed after suspension doses. In addition, the oral bioavailability of the solution doses was estimated in excess of 100% (Additional File 1: Table S4). Importantly, the AUC_inf_ of SJ311 at 200 mg/kg was equivalent to the AUC_inf_ at 100 mg/kg, but 5 times lower than that of SJ733. Collectively, these results are indicative of still sub-proportional, but more proportional dose-exposure relationships for (+)-SJ733, most likely due to saturable clearance as the dose is increased.

**Table 8:** Rat pharmacokinetic parameters of S733 and SJ311 after oral administration

		(+)-SJ733				(+)-SJ311			
Dose (mg/kg)		19.9 ± 0.2	50	100	200	20.9, 20.4^*a*^	50	100	200
Formulation		Solution	Suspension	Suspension	Suspension	Suspension	Suspension	Suspension	Suspension
Half life (h)		8.0 ± 0.6	7.7 ± 3.2	10.3 ± 6.9	7.7 ± 3.3	7.9, 7.9	3.5 ± 0.4	4.3 ± 2.2	4
C_max_ (µM)		11.6 ± 2.1	11.1 ± 1.2	16.6 ± 3.2	27.9 ± 3.8	4.7, 5.0	4.4 ± 2.0	7.1 ± 0.81	9.4 ± 5.4
T_max_ (h)		3.0 ± 0.9	4	3.0 ± 1.7	4.0 ± 3.5	1.0, 2.5	1.2 ± 0.8	4	3.2 ± 4.2
AUC_inf_ (h µM)		76.1 ± 5.6	96.3 ± 5.8	180 ± 23.2	330 ± 78.4	26.5, 27.1	34.1 ± 8.7	61.9 ± 11.5	65.8 ± 12.6
CL/F (L/h/kg)		ND	1.1 ± 0.1	1.2 ± 0.1	1.3 ± 0.3	1.6, 1.7	3.4 ± 1.0	3.6 ± 0.7	6.7 ± 0.5
V/F(L/kg)			12 ± 5.0	17 ± 9.0	15 ± 9.0	19.6, 18.7	17 ± 7.0	21 ± 8.0	38
F%		122 ± 10	74.5	69.6	63.8	38.0, 39.9	20.5	18.6	9.9
Dose in urine (%)		2.8 ± 1.0	ND		0.2 ± 0.1	14.9, 10.7	ND		1.1 ± 0.4

^a^n = 2; for all other PK studies, data are presented as mean ± SD (n = 3). AUC = AUC_inf_ and was used to calculate F%. ND = not determined

Finally, the pharmacokinetics of both compounds was examined following a single PO or IV administration to male beagle dogs, with an IV dose of 3 mg/kg (Additional file 1: Table S7) and PO doses of 3 and 30 mg/kg (Table 9). By the IV route, both compounds had roughly equivalent half-lives (8–10 h) but SJ311 exhibited a lower clearance and lower volume of distribution relative to SJ733. When dosed orally, SJ733 was more rapidly absorbed than SJ311 (1 vs 3 h T_max_). Both compounds exhibited similar half-lives at higher dose (~ 6 h) but SJ733 was more slowly eliminated at the lower dose (longer half-life), although there is higher variability in this factor that precludes definitive comment. Regardless of route, a significantly higher percentage (mean, 32–35% with high variability) of the SJ311 dose was eliminated in urine compared with that of SJ733 (2–3%). Both compounds had acceptable dose-proportionality and oral bioavailability (74–115%).

**Table 9:** Dog pharmacokinetic parameters of S733 and SJ311 after oral administration

Dose (mg/kg)	(+)-SJ733		(+)-SJ311	
	3	30	3	30
C_max_ (µM)	3.0 ± 0.7	25.6 ± 2.1	4.0 ± 0.8	35.0 ± 5.1
Tmax (h)	0.83	1.67	3.3 ± 1.2	3.0 ± 1.7
Half life (h)	11.4 ± 5.0	5.3 ± 0.7	6.1 ± 0.6	6.7 ± 1.4
AUC_inf_ (h.µM)	40.6 ± 12.9	243 ± 52.9	56.2 ± 12.1	601 ± 137
CL (L/h/kg)	0.169 ± 0.055	0.274 ± 0.067	0.121 ± 0.030	0.113 ± 0.025
V (L/kg)	2.6 ± 0.8	2.1 ± 0.8	1.1 ± 0.4	1.1 ± 0.4
F%	115 ± 29	73.7 ± 16.1	73.0 ± 15.8	77.9 ± 17.7
Dose in urine (%)	2.8 ± 0.2	3.1 ± 1.9	34.7 ± 15.9	33.4

Data are presented as mean ± SD (n = 3)

**Fig. 2:**
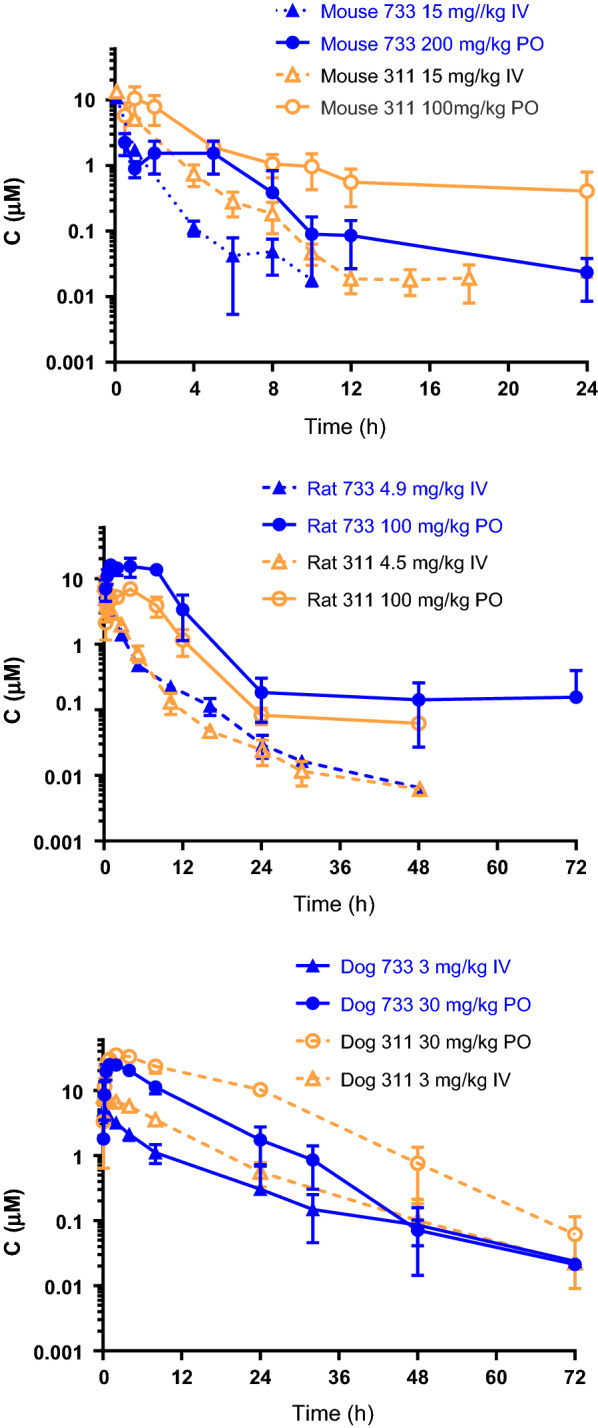
Pharmacokinetic profile of a single intravenous and oral dose of SJ733 and SJ311 in mouse, rat and dog. Racemic compounds were used for mouse IV/PO studies. Racemic SJ733 was used in rat IV study and active isomers were used in rat PO study. Only active isomers were used for rat studies for SJ311. Active isomers were used for all the dog studies. Animal number in each species: Mouse = 6, rat = 2 (IV), rat = 3 (PO), dog = 3

The plasma exposures of both compounds in all three species are summarized in Fig. 2. For easy comparison only one dose level per route of administration is presented. There was a modest correlation between in vitro intrinsic clearance pattern (Table 4) and the in vivo clearance in all three species. SJ733 exhibited higher in vitro clearance and in vivo clearance compared to SJ311 in mice. In rats and dogs, both compounds showed lower clearance, as expected based on the in vitro microsome studies.

### In vitro and in vivo correlation

The predicted in vivo blood clearance of SJ733 in mice (1.3–4.9 L/h/kg, units converted from data in Additional file 1: Table S2) based on data from mouse microsomes uncorrected for binding correlated well with the in vivo clearance (~ 3 L/h/kg) observed in mice (Table 7). The hepatocyte-predicted blood clearance (Additional file 1: Table S3, 2.0 ± 0.5 L/h/kg) in the rat (not corrected for binding) agreed well with the measured in vivo clearance (~ 0.7 L/h/kg) (Table 8) but over-predicted (three to tenfold) the in vivo clearance in mice and dogs (Additional file 1: Table S3, Table 9).

### Dose proportionality

**Fig. 3:**
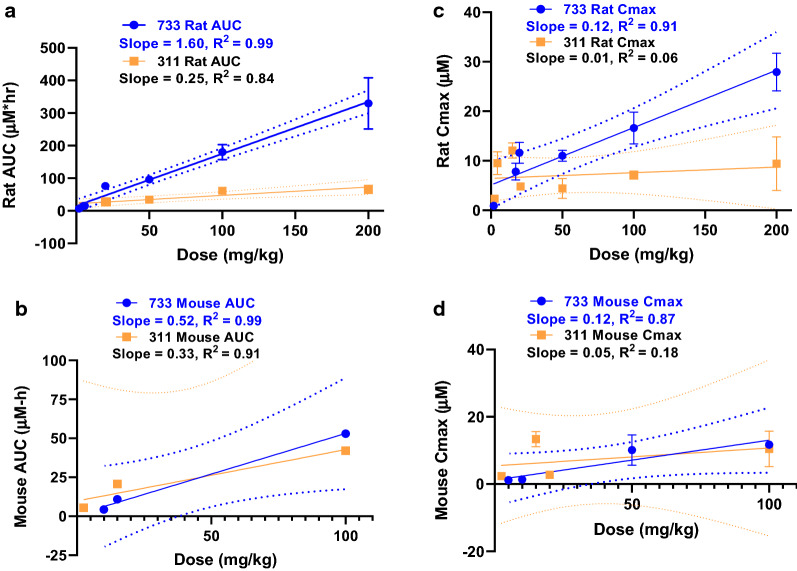
Dose proportionality of SJ733 and SJ311 as function of AUC_inf_ and C_max_, oral and intravenous dose combined**.** **a** Dose proportionality of SJ733 and SJ311 as function of AUC_inf_ in rats. **b** Dose proportionality of SJ733 and SJ311 as function of AUC_inf_ in mice. **c** Dose proportionality of SJ733 and SJ311 as function of Cmax in rats. **d** Dose proportionality of SJ733 and SJ311 as function of C_max_ in mice. Unweighted linear regression was performed with GraphPad Prism 8.0

The major difference between SJ311 and SJ733 was proportionality of dose-exposure relationships in rats, most evident at high doses. Linear regression analysis showed that SJ733 exposures were approximately dose proportional, whereas they appeared sub-proportional for SJ311 in both rats and mice. SJ733 had a narrower 95% confidence interval (95% CI) in total AUC_inf_ and dose- normalized C_max_ compared to SJ311 (Fig. 3).

## Discussion

Although malaria morbidity has steadily declined since peaking in the 2000s, the rate of that reduction plateaued in 2015–2018 [1]. Additionally, resistance has emerged to artemisinin co-therapies (ACT) [22, 23]. For these reasons, new small-molecule drugs remain a key strategic need for the malaria management. However, there are only two new chemical entities that received approvals since 2000 (combination of OZ277 and piperaquine, Krintafel/Kozensis containing tafenoquine). Therefore, the field requires improved methods to accelerate the discovery and development of malaria drugs. Any new drugs must work on multi-drug resistant *Plasmodium* species, be orally bioavailable, possess excellent safety profiles, and have pharmacokinetics consistent with requiring no more than three sequential daily doses.

The key data that drove the selection of SJ733 as a clinical candidate for malaria are presented. SJ733 is a member of the dihydroisoquinolone family (DHIQs). Extensive optimization of this class of molecules, including suppression of metabolism risk and improvement of physiochemical properties, led to two frontrunners: SJ311 and SJ733. They are identical except for the substitution of a pyridine ring (in SJ733) for a pyrazole (in SJ311). Both compounds possess similar pharmacology properties and potency. To select the best compound, the comparisons focused on the pharmacokinetics and bioavailability profiles across multiple species and wide dosing ranges, as these two factors remain the third most common cause of failure (16%) in phase I clinical trials [24].

After initial profiling using in vitro experiments, neither compound was a clear frontrunner. SJ311 possessed much lower solubility and permeability, both of which were independent of pH. This finding suggested absorption would be better for SJ733. However, SJ733 appeared to have much higher oxidative metabolism and thus was predicted to be more rapidly cleared in vivo.

While the change of pyridine to pyrazole prevented pyridine metabolism (N-oxidation), there was no significant difference in plasma exposure between SJ733 and SJ311 after a single IV bolus or oral administration in mice. The bioavailability of SJ311 in mice appeared much lower (22–23% vs. 65–81% for SJ733). SJ311 exhibited up to a 30-fold increase in renal elimination. Thus, renal clearance contributes to the elimination of SJ311 whereas SJ733 is mainly cleared through oxidative metabolism. Similar differences were seen in the dog. Lower partition coefficients, such as seen with SJ311, have previously been linked to higher renal clearance [16, 20]. Without bile secretion data, the exact mechanism of drug elimination is yet to be determined. Unbound drug molecules of less than 20,000 Da are filtered through the glomerulus with the primary urine. More significant renal reabsorption of SJ733 might explain the lower excretion in urine, as SJ733 has a higher LogD_7.4_ value than SJ311, and SJ733 is slightly less polar. Overall, while the mechanisms of disposition and metabolism were different, both compounds were cleared with similar rates.

Finally, a dose-exposure proportionality was examined in the rat. The AUC_inf_ of SJ311 plateaued at doses higher than 100 mg/kg in rats. On the other hand, the exposure of SJ733 was fivefold greater than that of SJ311 at the dose of 200 mg/kg and did not fully plateau at any studied dose. No adverse events were reported for SJ733 at any dose, with an AUC_inf_ over 217 µM h at the highest event free dose. Thus the provable therapeutic window for SJ733 was well above 40. The therapeutic index of SJ311 could be much lower and could not be proven to be larger than roughly sevenfold due to its relatively poor dose proportionality. Lack of dose proportionality of plasma exposure can be problematic in many aspects, including variable absorption, potential irritation to the GI tract, waste of compound, and drug tolerance. Based on all these considerations, SJ733 was prioritized over SJ311 for clinical development.

## Conclusion

There is still a high demand for developing new active ingredients to be used either as a single agent or in combination for malaria management. By carefully comparing two equipotent, structurally related ATP4 inhibitors, we were able to prioritize one, SJ733, based on a wider therapeutic window in preclinical toxicology species. This selection strategy using a range of pharmacokinetic and toxicokinetic studies enabled using PKPD modeling to support dose simulation in human. SJ733 has progressed into Phase 2a trials and the Phase 1 results have recently been reported.

## Supplementary Information

**Additional file 1: Table S1.** Summary of thermodynamic solubility data for ( +)-SJ733 obtained at 37 °C in phosphate buffer, 0.1 N HCl and simulated intestinal fluids. **Table S2.** Summary of measured CLint, *vitro* and scaled CLint, *vivo* of SJ733 and SJ311 (independent replicate experiments to Table 4). **Table S3.** Metabolic stability parameters for ( +)-SJ733 in human, dog, rat and mouse cryopreserved hepatocytes. **Table S4.** Rat pharmacokinetic study of S733 after oral administration. **Table S5.** Murine plasma pharmacokinetic parameters of S733 and SJ311 after intravenous injection. **Table S6.** Rat pharmacokinetic parameters of S733 and SJ311 after intravenous administration. **Table S7.** Dog pharmacokinetic parameters of S733 and SJ311 after intravenous administration.

## Abbreviations

ACT: Artemisinin-based combination therapies
MMV: Medicines for Malaria Venture
TPPs: Target Product Profiles
TCPs: Target Candidate Profiles
SJCRH: St Jude Children’s Research Hospital
IACUC: Institutional Animal Care and Use Committee
pKa: Ionization constant
LogD7.4: Logarithm of the octanol/pH 7.4 buffer partition coefficient
PBS: Phosphate buffered saline
PAMPA: Parallel Artificial membrane Permeability Assay
EMEM: Eagle's Minimum Essential Medium
HBSS: Hank's Balanced Salt Solution
HEPES: 4-(2-Hydroxyethyl)-1-piperazineethanesulfonic acid
FaSSIF: Fasted-state simulated intestinal fluid
FeSSIF: Fed-state simulated intestinal fluid
SGF: Simulated gastric fluid
CYP: Cytochrome P450
PBPK: Physiologically-based pharmacokinetic
CL_int_: Intrinsic clearance
PO: Oral gavage
IV: Intravenous
C_max_: Maximal plasma concentration
T_max_: Time to maximum plasma concentration
AUC: Area under the plasma concentration–time
AUC_inf_: Area under the plasma concentration–time curve extrapolated to infinity
t1/2: Terminal elimination half-life
Vss: Apparent volume of distribution at the steady state
CL: Systemic clearance
F: Oral bioavailability
