# Supplementary material for: Selecting an anti-malarial clinical candidate from two potent dihydroisoquinolones
Source: Malar J. 2021 Feb 19;20:107. doi: 10.1186/s12936-021-03617-1 (PMC7893776; doi:10.1186/s12936-021-03617-1)
Supplement: Supplementary file 1 — Additional file 1: Table S1. Summary of thermodynamic solubility data for ( +)-SJ733 obtained at 37 °C in phosphate buffer, 0.1 N HCl and simulated intestinal fluids. Table S2. Summary of measured CLint, vitro and scaled CLint, vivo of SJ733 and SJ311 (independent replicate experiments to Table 4). Table S3. Metabolic stability parameters for ( +)-SJ733 in human, dog, rat and mouse cryopreserved hepatocytes. Table S4. Rat pharmacokinetic study of S733 after oral administration. Table S5. Murine plasma pharmacokinetic parameters of S733 and SJ311 after intravenous injection. Table S6. Rat pharmacokinetic parameters of S733 and SJ311 after intravenous administration. Table S7. Dog pharmacokinetic parameters of S733 and SJ311 after intravenous administration. [file 12936_2021_3617_MOESM1_ESM.docx]

**Selecting an anti-malaria clinical candidate from two potent dihydroisoquinolones**

Yizhe Chen, Fangyi Zhu, Jared T. Hammill, Gloria Holbrook^b^, Lei Yang, Burgess B. Freeman, Karen L. White, David M. Shackleford, Kathleen G. O’Loughlin, Susan A. Charman, Jon C. Mirsalis and R. Kiplin Guy

**ADDITIONAL INFORMATION**

| **TABLE S1** | | | | | |
| --- | --- | --- | --- | --- | --- |
| **Summary of thermodynamic solubility data for (+)-SJ733 obtained at 37°C in phosphate buffer, 0.1 N HCl and simulated intestinal fluids** | | | | | |
| Media | pH | Solubility (µM)^a^ | | | |
|  |  | 1 h | 4 h | 6 h | 24 h |
| Phosphate buffer^b^ | 7.4 | 207 | 250 | 246^b^ | 250 |
| 0.1 N HCl | 1 | 6817 | 7321 | 7376 | 7891 |
| FeSSIF-V2 | 5.8 | 237 | 250 | 254^b^ | 248 |
| FeSSIF blank |  | 83 | 88 | 100 | 107 |
| FaSSIF-V2 | 6.5 | 199 | 216 | 216^b^ | 207 |
| FaSSIF blank |  | 81 | 92 | 96 | 100 |
| a Values represent the mean of duplicate technical replicates which differed by <15%, b ionic strength of 154 mM  b Data from Charman et al. (16) | | | | | |

| **TABLE S2** | | | | | | | | | |
| --- | --- | --- | --- | --- | --- | --- | --- | --- | --- |
| **Summary of measured CLint, *vitro* and scaled CLint, *vivo* of SJ733 and SJ311 (independent replicate experiments to Table 4)** | | | | | | | | | |
| Compound | Concentration (µM) | Mouse | | Rat | | Dog | | Human | |
|  |  | CLint*_in_* *_vitro_*^a^ | CLint*_in vivo_^b^* | CLint*_in_* *_vitro_* | CLint*_in vivo_* | CLint*_in_* *_vitro_* | CLint*_in vivo_* | CLint*_in_* *_vitro_* | CLint*_in vivo_* |
| (r)-SJ733 | 20 | <7 | < 13.5 | <7 | <11.7 | <7 | <8.3 | <7 | <5.2 |
|  | 4 | 10 ± 0.5 | 23.4 ± 1.1 | <7 | <11.7 | <7 | <8.3 | 11.5 ± 0.3 | 10.3 ± 1.2 |
|  | 0.8 | 9.1 ± 0.5 | 21.4 ± 1.3 | <7 | <11.7 | <7 | <8.3 | 12.7 ± 0.3 | 11.5 ± 0.3 |
| (-)-SJ733 | 20 | 10.5 ± 0.4 | 9.5 ± 3.5 | <7 | <11.7 | 6.0 ± 0.4 | 8.6 ± 0.6 | 25.3 ± 0.4 | 22.7 ± 0.3 |
|  | 4 | 26.9 ± 0.6 | 63.0 ± 2.1 | <7 | <11.7 | 9.5 ± 0.3 | 13.7 ± 0.5 | 40.4 ± 0.9 | 36.4 ± 0.8 |
|  | 0.8 | 30.6 ± 1.4 | 71.7 ± 3.4 | <7 | <11.7 | 9.8 ± 0.4 | 14.1 ± 0.5 | 52.4 ± 1.1 | 47.2 ± 1.0 |
| (+)-SJ733 | 20 | 15.9 ± 2.2 | 37.3 ± 5.3 | <7 | <11.7 | <7 | <8.3 | 20.6 ± 0.5 | 18.5 ± 0.4 |
|  | 4 | 27.2 ± 1.3 | 63.8 ± 2.9 | <7 | <11.7 | <7 | <8.3 | 34.5 ± 0.6 | 31.0 ± 0.5 |
|  | 0.8 | 33.9 ± 1.3 | 79.3 ± 3.0 | <7 | <11.7 | <7 | <8.3 | 42.6 ± 0.7 | 38.4 ± 0.7 |
| (r)-SJ311 | 20, 4, 0.8 | <7 | < 13.5 | <7 | <11.7 | <7 | <8.3 | <7 | <5.2 |
| ^a^ unit for CLint vitro: µl/min/mg protein. ^b^ unit for CLint vivo: ml/min/kg. Compounds with a calculated half-life more than 4 h were all reported as having a half-life of >4 h, and a clearance value < 7 µl/min/mg protein. ND: not determined. CLint *in vivo* was scaled up using the PBSF. | | | | | | | | | |

| **Metabolic stability parameters for (+)-SJ733 in human, dog, rat and mouse cryopreserved hepatocytes** | | | | | |
| --- | --- | --- | --- | --- | --- |
| Species | t_1/2_ | CLint*_in vitro_* | CLint*_in vivo_* | CLint*_in vivo_* | Predicted CL_blood_ |
|  | (h) | (µL/min/10^6^ viable cells) | (mL/min/kg) | (L/h/kg) | (mL/min/kg) |
| Mouse | 1.22, 1.08 | 28.4, 31.8 | 200, 224 | 12.0, 13.4 | 4.49, 4.69 |
| Rat | 1.82, 2.55 | 8.1, 5.8 | 38.1, 27.1 | 2.29, 1.63 | 1.64, 1.16 |
| Dog | 1.70, 1.72 | 6.5, 6.4 | 39.8, 39.5 | 2.39, 2.38 | 1.04, 1.04 |
| Human | 1.10, 1.05 | 13.6, 14.0 | 34.5, 35.6 | 2.07, 2.14 | 0.77, 0.79 |
| Compound were tested at 1 µM. Values represent duplicate measurements. | | | | | |

| **TABLE S4** | | | | | |
| --- | --- | --- | --- | --- | --- |
| **Rat pharmacokinetic study of S733 after oral administration** | | | | | |
|  | (r)-SJ733*^a^* | (+)-SJ733 | | | |
| Dose (mg/kg) | 22.8, 19.8 | 1.7 | 17.3 ± 0.2 | 1.9 *^b^* | 19.9 ± 0.2*^b^* |
| Half life (h) | 4.4, 4.7 | ND | 7.2 ± 1.5 | 11.3 ± 6.0 | 8.0 ± 0.6 |
| C_max_ (µM) | 7.2, 4.8 | 0.7 ± 0.1 | 7.8 ± 1.7 | 0.9 ± 0.1 | 11.6 ± 2.1 |
| T_max_ (h) | 1.0 | 0.7 ± 0.3 | 1.0 | 3.0 ± 0.9 | 3.0 ± 0.9 |
| AUC_inf_ (h.µM) | 24.1, 3.7 | 1.9 ± 0.6 *^c^* | 40.5 ± 2.0 | 6.6 ± 1.2 | 76.1 ± 5.6 |
| F% | 49, 79 | 36.7 ± 12 | 75.3 ± 3.2 | 111 ± 19 | 122 ± 10 |
| Dose in urine (%) | 0.5 | 0.5 ± 0.3 | 1.1 ± 0.1 | 1.2 ± 0.4 | 2.8 ± 1.0 |
| a n=2 for (r)-SJ733 oral PK in rats all other PK studies were mean ± SD (n=3). b Formulation was a solution, all others are suspensions. c AUC= AUC_0-tlast_ was used to calculate F%. Data are presented as mean ± SD (n=3). ND = not determined | | | | | |

|  | | |
| --- | --- | --- |
| **Table S5 Murine plasma pharmacokinetic parameters of S733 and SJ311 after intravenous injection** | | |
| Batch | (r)-SJ733*^a^* | (r)-SJ311*^b^* |
| Dose (mg/kg) | 15 | 15 |
| C_max_ (µM) | 11.3 | 13.4 |
| Half-life (h) | 1.52 | 1.56 |
| AUC_inf_ (h.µM) | 10.8 | 20.7 |
| CL (L/h/kg) | 2.98 | 1.63 |
| V_ss_ (L/kg) | 2.41 | 2.11 |
| a Average of 2 in vivo experiments. b the AUCi_nf_, CL and V_ss_ were estimated from mean plasma concentration values from different animals in a single study; error or SDs for the parameters were not estimated. | | |

|  | | | |
| --- | --- | --- | --- |
| **Table S6 Rat pharmacokinetic parameters of S733 and SJ311 after intravenous administration** | | | |
|  | (r)-SJ733 | (+)-SJ733*^a^* | (+)-SJ311 |
| Dose (mg/kg) | 5.0, 4.8 | 4.7 ± 0.1 | 4.5 |
| Half-life (h) | 5.2, 5.4 | 9.2 ± 0.4 | 18.7, 10.6 |
| AUC_inf_ (h.µM) | 11.2, 9.8 | 14.7 ± 0.7 | 17.4, 12.6 |
| CL (L/h/kg) | 0.95, 1.1 | 0.68 ± 0.02 | 0.56, 0.79 |
| V_ss_ (L/kg) | 5.4, 5.5 | 3.3 ± 0.2 | 2.2, 3.4 |
| Dose in urine (%) | 1.02, 0.74 | 1.6 ± 0.5 | 20.8, 23.2 |

a Average (± SD) of 3 in vivo experiments; n=2 for all other IV data.

|  | | |
| --- | --- | --- |
| **Table S7 Dog pharmacokinetic parameters of S733 and SJ311 after intravenous administration** | | |
|  | **(+)-SJ733** | (+)-SJ311 |
| Dose (mg/kg) | 3 | 3 |
| Half life (h) | 9.6 ± 4.7 | 7.8 ± 2.2 |
| AUC_inf_ (h.µM) | 33.1 ± 10.0 | 77.0 ± 12.4 |
| CL (L/h/kg) | 0.204 ± 0.054 | 0.085 ± 0.013 |
| V_ss_ (L/kg) | 2.8 ± 1.8 | 0.96 ± 0.27 |
| Dose in urine (%) | 1.9 ± 0.3 | 32.3 ± 21.3 |
| Data are presented as mean ± SD (n= 3) | | |
